# Influence of increased heart rate and aortic pressure on resting indices of functional coronary stenosis severity

**DOI:** 10.1007/s00395-017-0651-0

**Published:** 2017-09-13

**Authors:** Lorena Casadonte, Bart-Jan Verhoeff, Jan J. Piek, Ed VanBavel, Jos A. E. Spaan, Maria Siebes

**Affiliations:** 1 Department of Biomedical Engineering and Physics, Academic Medical Center, University of Amsterdam, PO Box 22660, 1100 DD Amsterdam, The Netherlands; 2 Department of Cardiology, Academic Medical Center, University of Amsterdam, Amsterdam, The Netherlands; 3 Present Address: Department of Internal Medicine, St Jansdal Hospital, Harderwijk, The Netherlands

**Keywords:** Coronary blood flow, Coronary artery stenosis, Metabolic adaptation, Baseline stenosis indices, Microvascular resistance

## Abstract

Baseline assessment of functional stenosis severity has been proposed as a practical alternative to hyperemic indices. However, intact autoregulation mechanisms may affect intracoronary hemodynamics. The aim of this study was to investigate the effect of changes in aortic pressure (Pa) and heart rate (HR) on baseline coronary hemodynamics and functional stenosis assessment. In 15 patients (55 ± 3% diameter stenosis) Pa, intracoronary pressure (Pd) and flow velocity were obtained at control, and during atrial pacing at 120 bpm, increased Pa (+30 mmHg) with intravenous phenylephrine (PE), and elevated Pa while pacing at sinus heart rate (PE + sHR). We derived rate pressure product (RPP = systolic Pa × HR), baseline microvascular resistance (BMR = Pd/velocity), and stenosis resistance [BSR = (Pa − Pd)/velocity] as well as whole-cycle Pd/Pa. Tachycardia (120 ± 1 bpm) raised RPP by 74% vs. control. Accordingly, BMR decreased by 27% (*p* < 0.01) and velocity increased by 36% (*p* < 0.05), while Pd/Pa decreased by 0.05 ± 0.02 (*p* < 0.05) and BSR remained similar to control. Raising Pa to 121 ± 3 mmHg (PE) with concomitant reflex bradycardia increased BMR by 26% (*p* < 0.001) at essentially unchanged RPP and velocity. Consequently, BSR and Pd/Pa were only marginally affected. During PE + sHR, velocity increased by 21% (*p* < 0.01) attributable to a 46% higher RPP (*p* < 0.001). However, BMR, BSR, and Pd/Pa remained statistically unaffected. Nonetheless, the interventions tended to increase functional stenosis severity, causing Pd/Pa and BSR of borderline lesions to cross the diagnostic threshold. In conclusion, coronary microvascular adaptation to physiological conditions affecting metabolic demand at rest influences intracoronary hemodynamics, which may lead to altered basal stenosis indices used for clinical decision-making.

## Introduction

Traditional indices for the functional assessment of coronary artery stenosis severity are derived from invasive intracoronary measurements obtained at maximal hyperemia. More recently, non-hyperemic indices obtained during contrast-induced reactive hyperemia [16, 27] or at resting coronary blood flow [23, 35, 37] have been introduced to facilitate functional stenosis assessment by obviating the need for vasodilator agents. Pressure-derived baseline indices include the distal coronary-to-aortic pressure ratio calculated over the entire cardiac cycle (Pd/Pa) [15, 23] or during a “wave-free” period in diastole, the instantaneous wave-free ratio (iFR) [1, 35]. In addition, basal stenosis resistance (BSR) has been proposed, defined as the ratio of the stenosis pressure gradient to resting flow velocity [37].

Although baseline indices have shown a strong correlation with the corresponding hyperemic values, the diagnostic accuracy suffers from a larger “gray zone” around the binary cut-off values for decision-making [15]. To improve diagnostic efficiency, while at the same time reducing the need for vasodilator drugs, hybrid strategies have been suggested, whereby hyperemia is only induced when measurements at resting flow fall within a certain gray zone [6, 7, 15, 30, 32].

Physiologically, coronary blood flow at rest is well regulated and adapted to metabolic demand [5, 14]. We hypothesized that variations in resting coronary blood flow in response to altered systemic pressure and myocardial oxygen consumption would affect resting indices of coronary stenosis severity. Accordingly, the aim of the present study was to investigate the influence of changes in heart rate and systemic pressure on physiological indices of stenosis severity, Pd/Pa and BSR, obtained in the presence of functional flow control mechanisms.

## Methods

### Patient characteristics

This is a single-center, retrospective study, which enrolled patients with stable coronary artery disease and a single de novo lesion in a coronary vessel scheduled for elective percutaneous coronary intervention. Clinical exclusion criteria were diffuse or three-vessel disease, ejection fraction below 30%, recent myocardial infarction, serious valve abnormalities, prior cardiac surgery, hypertrophic cardiomyopathy, cardiac arrhythmia, abnormal clotting profiles, or severe renal failure.

### Cardiac catheterization and hemodynamic measurements

All anti-anginal medication was continued. A 5F- or 6F-guiding catheter was introduced via standard femoral approach for cardiac catheterization. Intracoronary nitroglycerin (0.1 mg) was administered prior to diagnostic angiography and repeated if the procedure lasted more than 30 min. Distal pressure (Pd) and Doppler flow velocity were simultaneously measured downstream of the stenosis (ComboWire, Volcano Corp., Rancho Cordova, CA, USA). The pressure transducer was normalized to aortic pressure at the ostium prior to obtaining distal measurements, and care was taken to optimize the quality of the flow velocity signal. Potential pressure offsets were checked at the ostium at the end of the procedure. A 6F bipolar pacemaker lead was placed into the right atrium. All signals were digitally recorded together with the ECG on a personal computer after 12-bit A/D conversion at 120 Hz, as previously described [36].

### Experimental protocol

Intracoronary hemodynamic signals were collected during a stable period at baseline (control) and after 2 min of atrial pacing at 120 bpm (Pac). After return to baseline, Pa was elevated by slow intravenous infusion of phenylephrine (PE) at an initial rate of 12 mg/min, adjusted upwards over a period of several minutes to reach a target increase in mean arterial pressure of 30 mmHg. Measurements at elevated pressure were repeated while pacing at sinus heart rate (PE + sHR) to counteract the reflex bradycardia during phenylephrine infusion.

### Data analysis

Angiograms were quantitatively analyzed to yield study vessel dimensions and stenosis diameter reduction (QCA-CMS 5.2, Medis Medical Imaging Systems, Leiden, Netherlands). For each protocol step, we estimated oxygen consumption by the rate pressure product (RPP) as the product of peak systolic Pa and HR. Cycle-averaged values were obtained for all hemodynamic variables based on ECG R-peaks. We corrected for pressure drift by means of the zero-flow intercept extrapolated from the stenosis pressure drop–velocity relationship at the time of stenosis assessment, as done previously [27, 37]. Diastolic values of pressure and velocity measurements were determined as the average from the aortic pressure dicrotic notch to the ECG R-peak. Whole-cycle and diastolic Pd/Pa were derived as pressure-only indices of stenosis severity. We determined BSR as the whole-cycle ratio of the stenosis pressure gradient (Δ*P*) to flow velocity and baseline microvascular resistance (BMR) as the ratio of Pd to flow velocity. Results represent the mean value over eight consecutive cycles.

### Statistical analysis

Continuous data are expressed as mean ± SEM. Normal distribution was checked with Shapiro–Wilk statistics. Effects of medication or risk factors on intrinsic resting flow velocity were assessed by multinomial logistic regression. Continuous variables at the patient level were compared between protocol steps using repeated measures analysis of variance followed by Tukey post hoc contrast analysis. Associations between variables at control and after hemodynamic provocation were assessed by linear regression analysis. A linear mixed-effects model with intervention as repeated effect at three levels was used to examine associations across all imposed interventions. Linear regression analysis with the models (Pd/Pa)_intervention_ = *A* × (Pd/Pa)_control_ + (1 − *A*) and (BSR)_intervention_ = *B* × (BSR)_control_ was performed to investigate the effect of functional stenosis severity on the change during the interventions from respective control values. These models satisfy the condition of Pd/Pa = 1 and BSR = 0 in case of no stenosis. The respective ischemic thresholds for Pd/Pa ≤0.92 [15] and BSR >0.66 mmHg cm^−1^ s [37] were used for diagnostic classification. Statistical tests were performed using SPSS vs. 20 (IBM, Armonk, NY, USA). Two-tailed values of *p* < 0.05 were considered statistically significant.

## Results

### Patient characteristics

Nineteen patients participated in this study. No patient experienced adverse clinical events or ischemic episodes during execution of the protocol. In three patients, it was not possible to perform the last step of the protocol due to patient discomfort related to the duration of the procedures. One patient was excluded from the analysis due to sub-optimal flow signal quality. Therefore, the study population consisted of 15 patients with a stenosis of intermediate severity (32–74% diameter reduction, reference diameter 3.06 ± 0.18 mm), predominantly located in the left anterior descending artery. Patient demographics and angiographic findings are summarized in Table 1.

**Table 1:** Demographics and stenosis characteristics (*n* = 15)

Age (years)	57 ± 2
Male sex	13 (87)
Diameter reduction (%)	55 ± 3
Study vessel LAD/LCX/RCA	12/2/1 (80/13/7)
Prior myocardial infarction	2 (13)
Coronary risk factors	
Hypertension	6 (40)
Smoking	6 (40)
Hypercholesterolemia	8 (53)
Diabetes	0 (0)
Medication	
ACE inhibitors	3 (20)
Aspirin	14 (93)
β-Blockers	10 (67)
Calcium antagonist	8 (53)
Nitrates	4 (27)

Values are expressed as mean ± SEM or *n* (%)

*ACE* angiotensin-converting enzyme, *LAD* left anterior descending artery, *LCX* left circumflex artery, *RCA* right coronary artery

### Induced changes in systemic and coronary hemodynamics

Hemodynamic variables and derived indices for all protocol steps are summarized in Table 2.

**Table 2:** Hemodynamic variables and derived coronary and stenosis indices at baseline over the entire cardiac cycle and during diastole

	Ctrl		Pac		PE		PE + sHR	
	Whole-cycle	Diastole	Whole-cycle	Diastole	Whole-cycle	Diastole	Whole-cycle	Diastole
Heart rate (bpm)	68 ± 3	–	120 ± 1^†^	–	52 ± 3^†,§^	–	68 ± 3^§^	–
Pa (mmHg)	96 ± 3	89 ± 2^¶^	105 ± 3^†^	100 ± 3^†,¶^	121 ± 3^†§^	109 ± 3^†,§,¶^	135 ± 4^†,§^	121 ± 4^†,§,¶^
Pd (mmHg)	87 ± 3	76 ± 3^¶^	91 ± 4	75 ± 5^¶^	110 ± 4^†,§^	94 ± 4^†,§,¶^	120 ± 5^†,§^	98 ± 6^†,¶^
∆P (mmHg)	9 ± 2	13 ± 3^II^	15 ± 3*	25 ± 5^#,¶^	11 ± 2^ǂ^	15 ± 3^§,II^	14 ± 3*^,ǂ^	22 ± 4^#,§,II^
Velocity (cm/s)	14 ± 1	17 ± 2^II^	19 ± 2^†^	24 ± 2^†,II^	13 ± 1^§^	15 ± 1^§,¶^	17 ± 1*^,§^	21 ± 2*^,§,¶^
BMR (mmHg cm^−1^ s)	7.04 ± 0.63	N/A	5.18 ± 0.41*	N/A	9.05 ± 0.69*^,§^	N/A	7.60 ± 0.61^§^	N/A
Pd/Pa	0.91 ± 0.02	0.85 ± 0.03^¶^	0.86 ± 0.03^#^	0.75 ± 0.05^#,¶^	0.91 ± 0.02^ǂ^	0.86 ± 0.03^§,II^	0.89 ± 0.02	0.81 ± 0.04^ǂ,II^
BSR (mmHg cm^−1^ s)	0.72 ± 0.17	N/A	0.82 ± 0.17	N/A	0.89 ± 0.20	N/A	0.93 ± 0.25	N/A

Values are expressed as mean ± SEM

*BMR* baseline microvascular resistance, *BSR* baseline stenosis resistance, *Ctrl* control, ∆*P* pressure gradient; *Pa* aortic pressure, *Pac* pacing, *Pd* distal pressure, *PE* phenylephrine, *PE* *+* *sHR* phenylephrine + pacing at sinus heart rate, *N/A* not applicable

* p < 0.05, ^#^ *p* < 0.01, ^†^ *p* < 0.001 vs. Ctrl; ^ǂ^ *p* < 0.05, ^§^ *p* < 0.01 vs. prior step; ^II^ *p* < 0.01, ^¶^ *p* < 0.001 vs. whole-cycle value

**Fig. 1:**
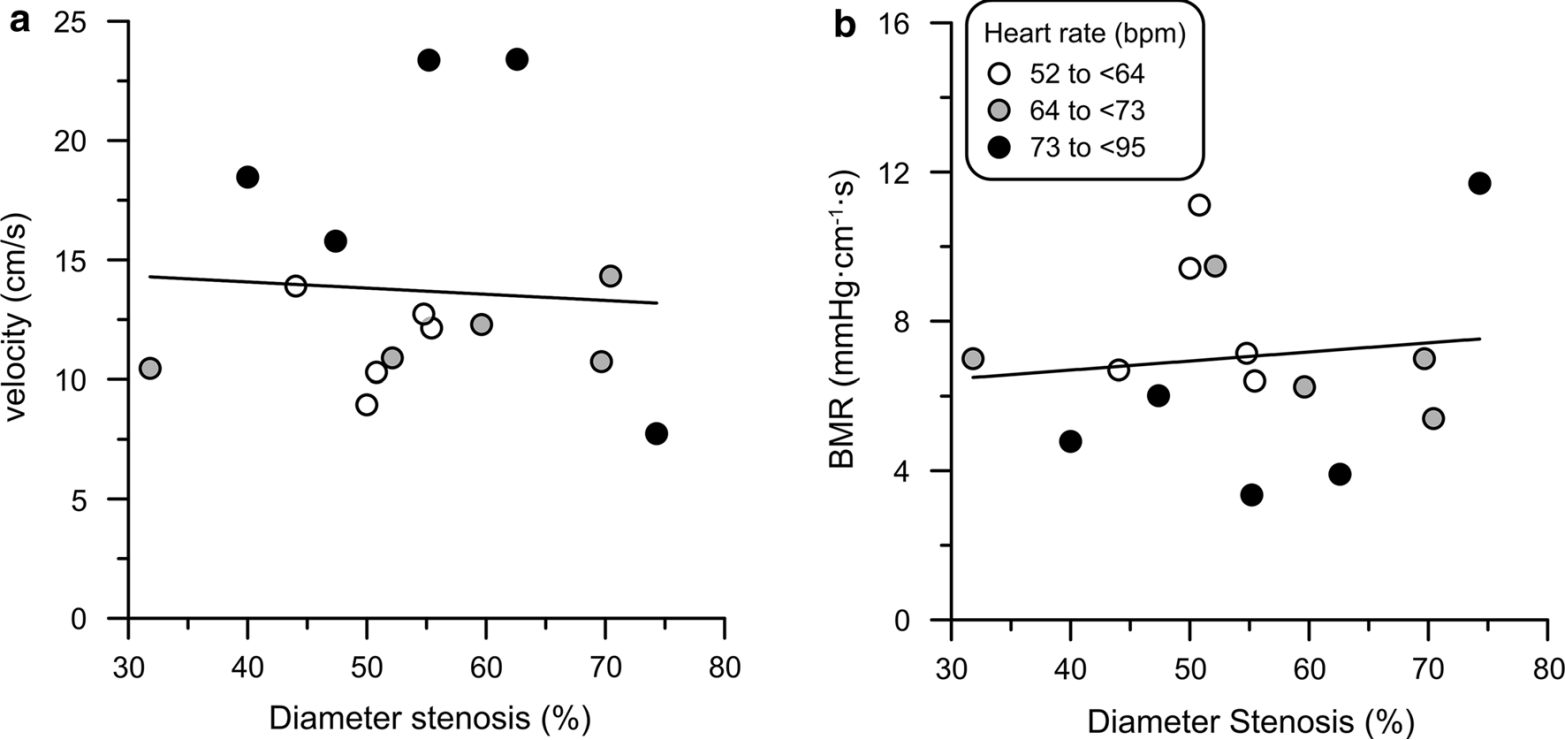
Resting blood flow velocity and baseline microvascular resistance (BMR) at control vs. anatomical stenosis severity. Flow velocity (**a**) or BMR (**b**) was not related to diameter stenosis. However, the average heart rate differed significantly for data points above the regression line compared with those below (*p* < 0.01)

**Fig. 2:**
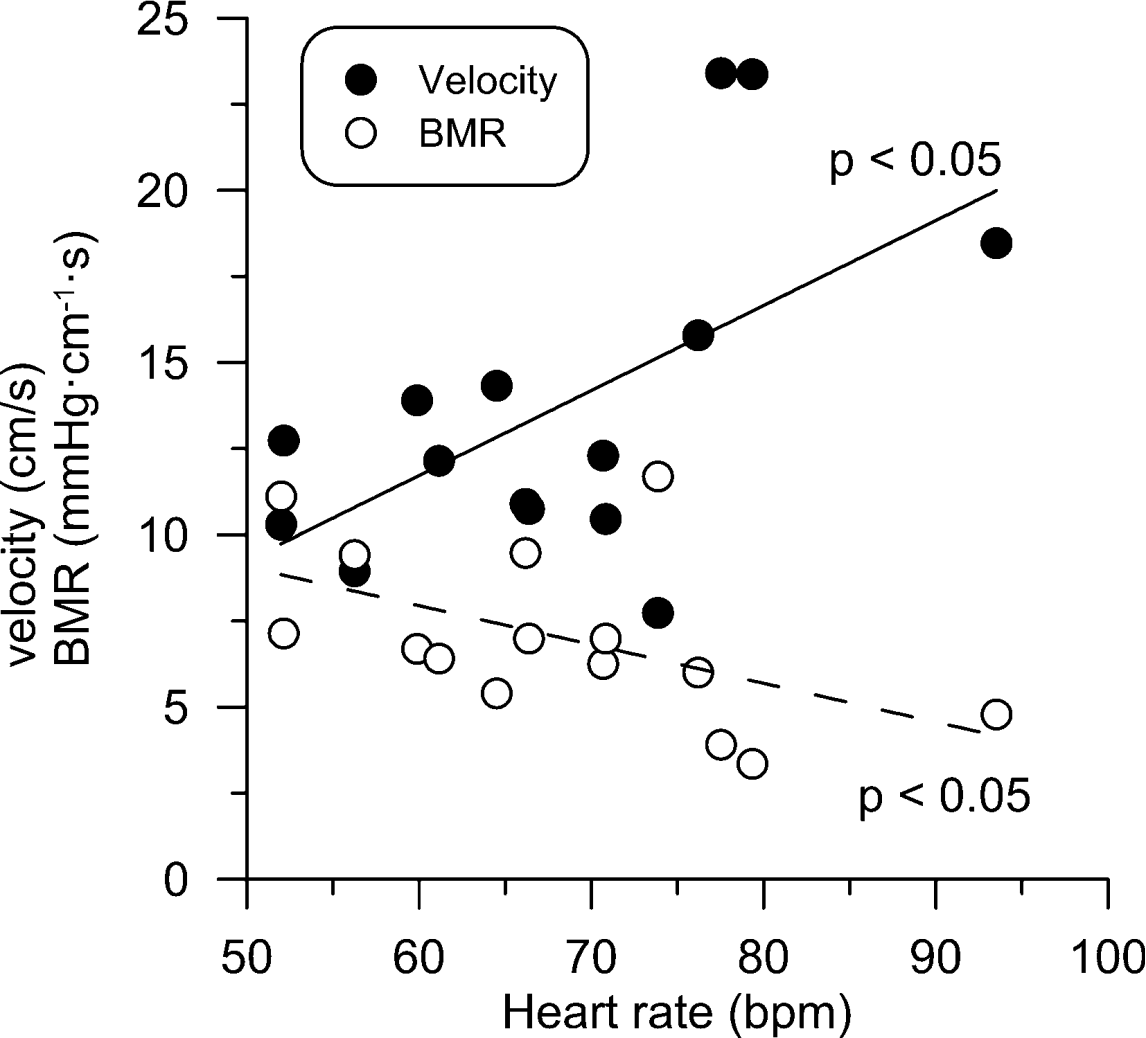
Dependence of resting flow velocity and baseline microvascular resistance (BMR) on heart rate at control. Coronary flow velocity increased significantly with resting HR, whereas BMR decreased

At control, Pa ranged from 79 to 121 mmHg, Pd from 73 to 114 mmHg, and heart rate from 52 to 94 bpm. Resting flow velocity ranged from 7.7 to 23.4 cm/s, with no difference due to medication or risk factors. In addition, BMR varied by a factor of three, ranging from 3.4 to 11.7 mmHg cm^−1^ s, and was strongly inversely related to flow velocity (*r* = −0.85, *p* < 0.001). BMR or resting velocity had no significant relationship with stenosis diameter reduction (Fig. 1). Of note, the average heart rate for velocity data above the regression line was 78 ± 5 compared with 63 ± 2 bpm for those below, and vice versa for BMR (*p* < 0.01). Both resting flow velocity (*r* = 0.58, *p* < 0.05) and BMR (*r* = −0.52, *p* < 0.05) were significantly associated with heart rate at control (Fig. 2).

Rapid atrial pacing increased heart rate by 52 ± 3 bpm (76%) and raised Pa by 9 ± 2 mmHg (9%) (both *p* < 0.001), while Pd remained essentially unchanged (*p* = 0.15). Resting coronary flow velocity increased by 5 ± 1 cm/s (36%, *p* < 0.01) and Δ*P* by 6 ± 2 mmHg (67%, *p* < 0.01). BMR decreased by 1.9 ± 0.4 mmHg cm^−1^ s (−27%, *p* < 0.01).

Phenylephrine infusion elevated Pa by 25 ± 2 mmHg (26%) vs. control (*p* < 0.001) with a concomitant reflex decrease in HR by 16 ± 1 bpm (−24%, *p* < 0.001). Coronary flow velocity and Δ*P* remained equivalent to control (*p* = 0.43 and *p* = 0.24, respectively), while Pd increased by 23 ± 3 mmHg (26%, *p* < 0.001) and BMR by 2.0 ± 0.7 mmHg cm^−1^ s (28%, *p* < 0.05).

Additional pacing at sinus heart rate further increased Pa to 38 ± 3 mmHg (40%) above control (*p* < 0.001). Distal flow velocity increased by 3 ± 1 cm/s (21%) and Pd by 33 ± 4 mmHg (+38%) vs. control (both *p* < 0.01), with a 5 ± 2 mmHg (56%) increase in Δ*P* (*p* < 0.01). BMR decreased by 1.5 ± 0.2 mmHg cm^−1^ s (−17%, *p* < 0.001) vs. PE, to a similar level as in control (*p* = 0.43).

**Fig. 3:**
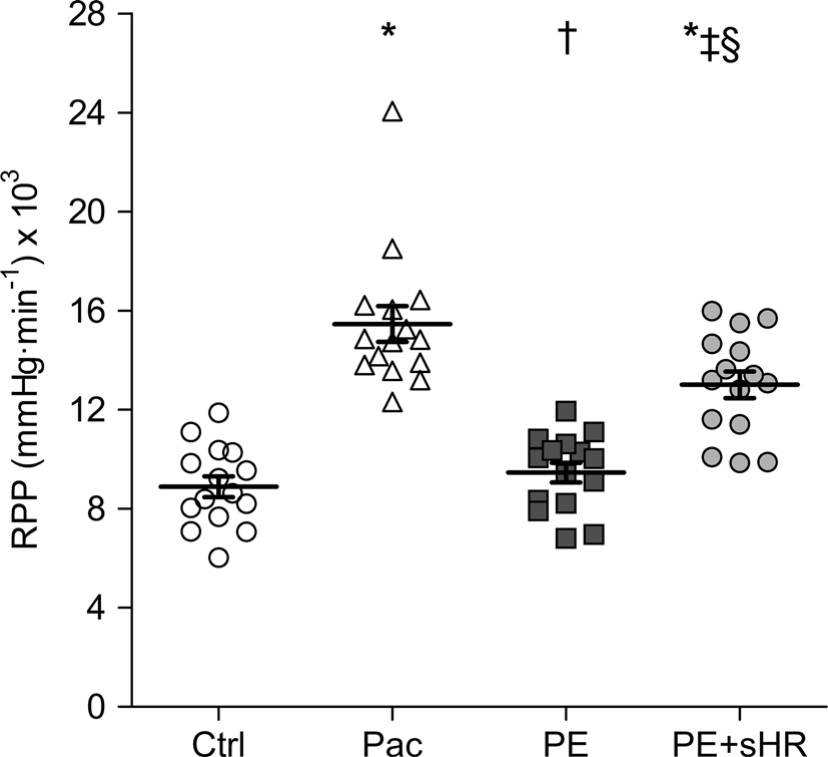
Rate pressure product (RPP) during different stages of the protocol. RPP did not change from control (Ctrl) during elevated aortic pressure (PE) with reflex bradycardia, but increased by 74 and 46% during pacing (Pac) and elevated Pa at sinus heart rate (PE + sHR), respectively. *p < 0.001 compared with control, ^†^ *p* < 0.001 and ^‡^ *p* < 0.01 compared with Pac, ^§^ *p* < 0.001 compared with PE

As shown in Fig. 3, RPP tended to increase slightly above control levels during PE (6%, *p* = 0.07), whereas Pac and PE + sHR substantially raised RPP by 74 and 46%, respectively (both *p* < 0.001).

**Fig. 4:**
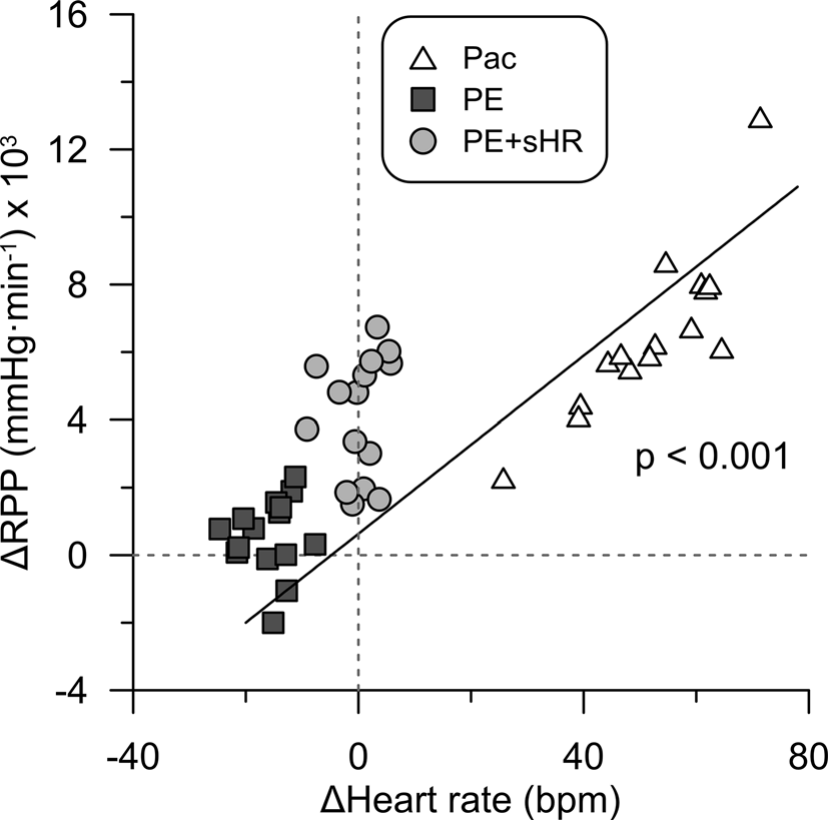
Effect of changes in heart rate on myocardial oxygen consumption. Changes in heart rate from control were positively associated with changes in rate pressure product (RPP). *Pac* pacing, *PE* phenylephrine, *PE* *+* *sHR* PE while pacing at sinus heart rate

**Fig. 5:**
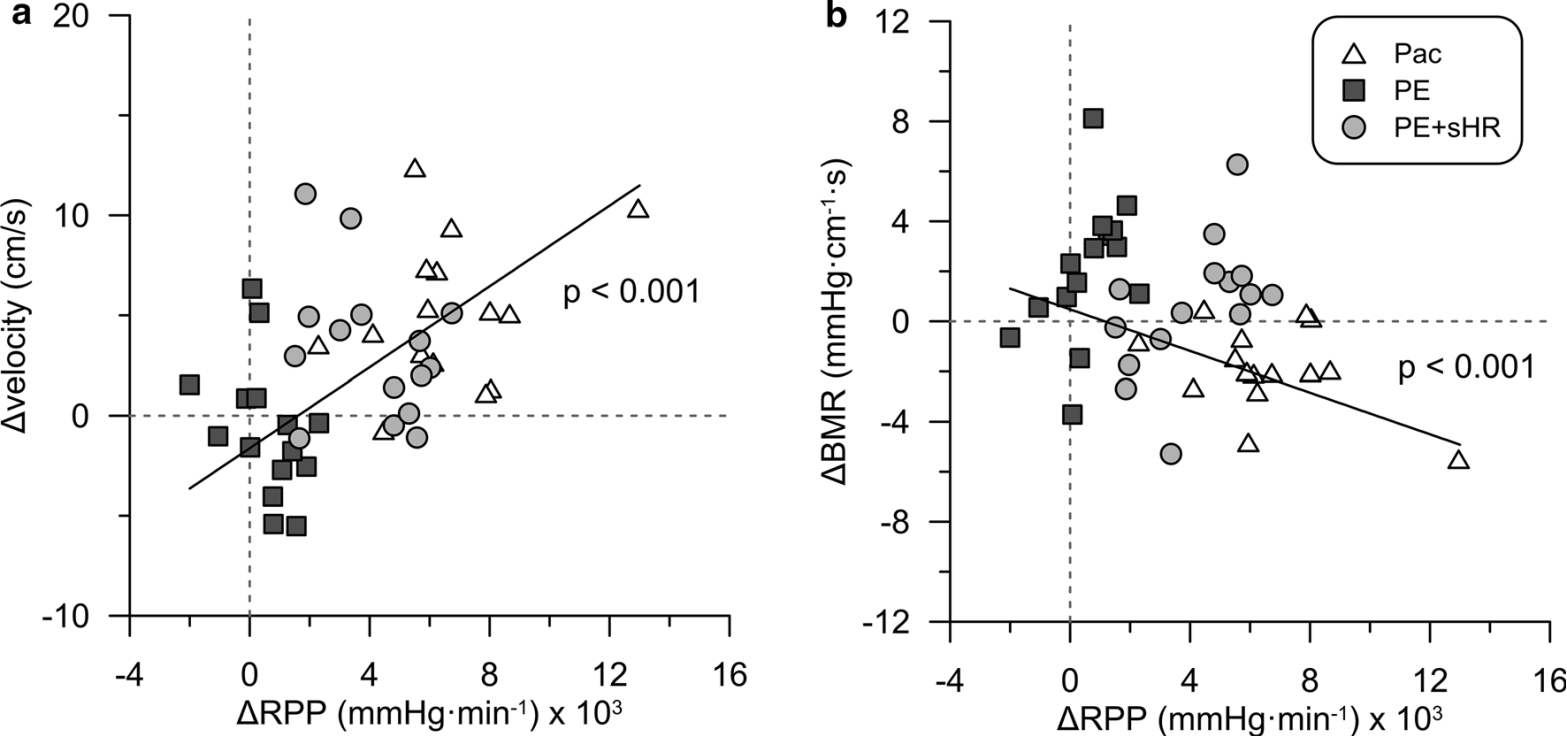
Effect of changes in oxygen consumption on changes in coronary flow velocity and baseline microvascular resistance (BMR). Changes in rate pressure product (RPP) from control across all interventions were **a** positively associated with changes in coronary flow velocity and **b** inversely with changes in BMR

Figure 4 illustrates that changes in RPP from control were strongly associated with changes in HR across all interventions (*p* < 0.001). Accordingly, changes in flow velocity and BMR (Fig. 5) during the hemodynamic provocations were strongly related to changes in RPP (both *p* < 0.001).

Overall, diastolic and whole-cycle results (Table 2) changed in the same direction in response to altered conditions. Although diastolic velocity and Δ*P* were higher compared with whole-cycle averages, non-significant changes vs. control observed for whole-cycle averages remained non-significant also for diastolic averages.

### Induced changes in basal functional indices

On average, the intra-patient whole-cycle decrease in Pd/Pa from control during pacing was 0.05 ± 0.02 (*p* < 0.01), while no significant change was observed during PE (*p* = 0.97) or PE + sHR (*p* = 0.21). Diastolic Pd/Pa was lower for each condition compared with whole-cycle Pd/Pa (*p* < 0.001 for Ctrl and Pac, *p* < 0.01 for PE and PE + sHR), and showed the same behavior for changes from control. Although BSR increased from control by 0.10 ± 0.07, 0.17 ± 0.12, and 0.22 ± 0.14 mmHg cm^−1^ s during Pac, PE, and PE + sHR, respectively, statistical significance was not reached (Table 2).

**Fig. 6:**
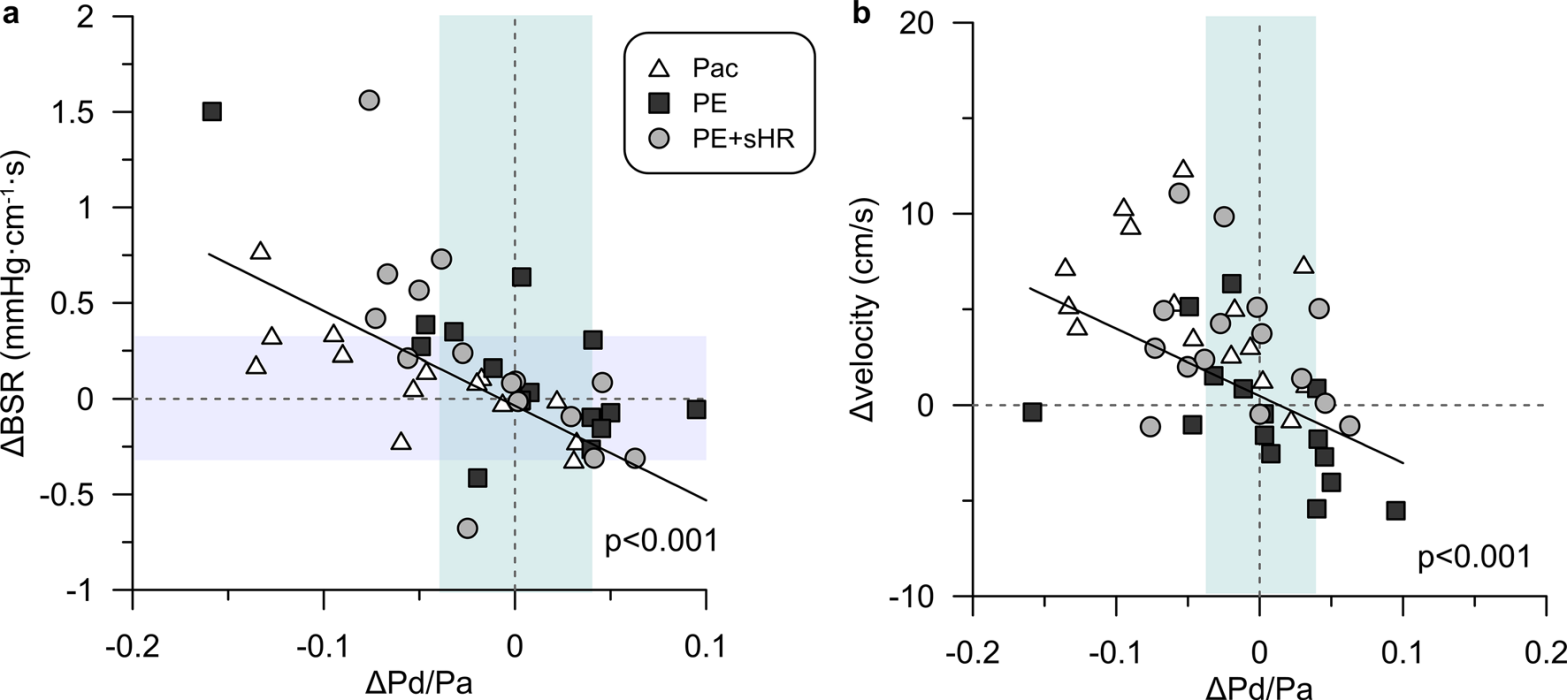
Individual changes **a** in basal stenosis resistance (BSR) and **b** in baseline flow velocity vs. changes in whole-cycle Pd/Pa induced by metabolic adaptation during the interventions. These changes exceeded the extent of the respective non-ischemic range for each index, as indicated by shaded areas for reference purposes. A significant negative correlation to changes in Pd/Pa across all interventions was found for both the change in BSR and change in flow velocity

Changes from control in both BSR (Fig. 6a) and velocity (Fig. 6b) during the interventions were negatively correlated with changes in Pd/Pa. To put the observed variations in baseline indices resulting from altered heart rate and aortic pressure into diagnostic perspective, we normalized these to the respective non-ischemic decision margin for BSR (0.66 mmHg cm^−1^ s) and Pd/Pa (0.08 = 1–0.92). The average normalized change during Pac, PE, and PE + sHR was, respectively, 15, 26, and 33% of the non-ischemic range for BSR, and −58, 1 and −19% for Pd/Pa.

**Fig. 7:**
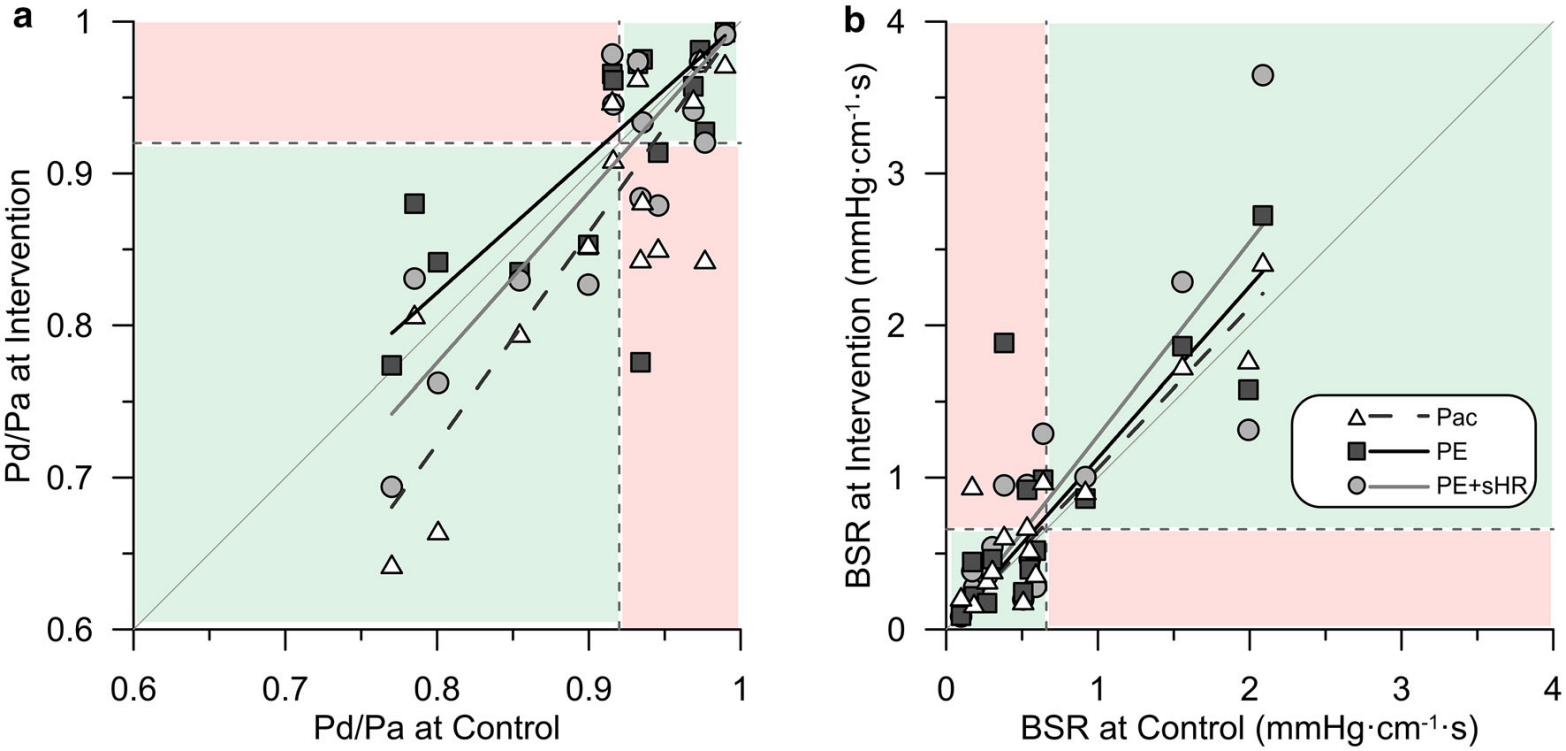
Comparison of baseline stenosis indices during hemodynamic provocation vs. control. The respective diagnostic threshold is indicated by the dashed lines. **a** Model-based regressions highlight the progressively lowering effect of pacing on Pd/Pa with increasing stenosis severity. Four lesions became functionally significant and one lost significance during pacing, while two lesions each crossed the threshold in opposite directions during the other interventions involving elevated aortic pressure. **b** BSR tended to increase during the hemodynamic provocations, with less dependence on functional lesion severity at control. Three lesions switched to a diagnostically significant BSR value during each intervention. Shaded areas indicate where the clinical classification agreed (green) or disagreed (red) between control and interventions. *Pac* pacing, *PE* phenylephrine, *PE* *+* *sHR* PE while pacing at sinus heart rate, *Pd/Pa* distal-to-aortic pressure ratio, *BSR* baseline stenosis resistance

Figure 7 shows the relation between pairs of values for Pd/Pa and BSR obtained before and after the hemodynamic interventions. Model-based regression analysis (Fig. 7a) demonstrated that especially tachycardia had a progressively lowering effect on Pd/Pa with increasing physiological stenosis severity at control (*r* = 0.81, *p* < 0.01). Similar results were obtained for diastolic Pd/Pa. In contrast, despite a worsening trend, changes in BSR (Fig. 7b) were much less related to its value at control.

Nonetheless, several borderline lesions switched diagnostic classification during hemodynamic provocation, with an overall worsening tendency. Pd/Pa became functionally significant during pacing tachycardia for four lesions (27%), and not significant for one (7%), while 2 lesions each (13%) crossed the Pd/Pa threshold in opposite directions both during PE and PE + sHR (Fig. 7a). Each of the hemodynamic provocations caused the BSR of three lesions (20%) to become functionally significant (Fig. 7b). Two patients in which Pd/Pa became diagnostically significant during pacing were the same as those in which BSR became significant.

Changes in diagnostic classification for diastolic Pd/Pa could not be assessed, since there is no established threshold for diastolic Pd/Pa as we calculated it.

## Discussion

In this study, we altered aortic pressure and heart rate at resting conditions to evaluate the dependence of baseline indices of functional stenosis severity on hemodynamic provocations in the presence of a functional autoregulation. The main findings are: (1) resting flow velocity and BMR at control were related to heart rate, rather than anatomical stenosis severity. (2) BMR and, consequently, velocity adapted as a physiological response to prevailing myocardial metabolic demand. (3) Basal indices of stenosis severity were differentially affected depending on concurrent changes in coronary flow velocity and distal pressure. Tachycardia had the largest effect, especially on Pd/Pa. (4) For about 20% of borderline lesions, BSR and Pd/Pa crossed their respective diagnostic threshold to a physiologically significant classification during the interventions.

### Effect of Pa and HR on basal coronary hemodynamics

The functional assessment of stenosis severity at baseline is an attractive option from a procedural point of view, since it does not require pharmacological vasodilation. Important physiological principles concerning resting indices are the recruitment of coronary dilatory capacity to adapt coronary blood flow to metabolic demand and autoregulatory compensation for reduced post-stenotic pressure [5, 14]. Coronary autoregulation is the result of a complex interaction of various mechanisms active at different levels of resistance vessels in the coronary tree. All arteries with diameter <400 µm contribute to the control of blood flow and autoregulation. A change in metabolic state is coupled via several mechanisms to microvascular function (resistance) and multiple neuro-humoral factors modulate microvascular control (e.g. α-agonist vasomotion and β-adrenergic increase in metabolic demand) [5, 12]. In addition, pathological syndromes affect regulation of resistance vessels depending on their diameter [9, 10, 29]. Resting coronary blood flow per myocardial mass is mainly related to oxygen consumption, which in turn depends on major factors such as heart rate and cardiac workload [5, 14]. This may well introduce considerable variability according to the actual metabolic state of individual patients. Modulation of resting coronary flow has also been reported due to circadian variation in sympathetic activity or via sympathetic stimulation during catheterization procedures [8, 25]. However, such biological variability has not been taken into account when assessing resting indices of stenosis severity and their diagnostic accuracy.

A major justification for pressure-based resting indices derives from the presumption of constant resting flow velocity regardless of stenosis severity. Yet, such generalization of constant basal flow velocity in fact implies a similar statement on constant metabolic demand for all interrogated patients. The average resting flow velocity was recently reported to be constant across a wide spectrum of stratified stenosis severities [26], whereas the stated variation around the mean may well reflect effects of heart rate for individual patients, which unfortunately was not reported or considered in that study. Our pre-intervention findings show that baseline velocity was not constant across all stenosis severities, but varied with the intrinsic patient-specific heart rate at control. Significant relationships were found between coronary flow velocity and BMR, as well as between BMR or velocity and heart rate, hence contrasting with those earlier findings [26].

Imposing controlled metabolic stressors elicited individual responses resulting in additional variability. Consistent with the previous findings in dogs [13, 19] and humans [24, 34, 39, 40], tachycardia increased oxygen consumption, inducing commensurate arteriolar vasodilation and a consequential increase in coronary flow velocity, while raising coronary distending pressure caused an increase of BMR as an autoregulatory response. Yet, one would expect some metabolic vasodilation because of increased cardiac afterload [2], which, however, was offset by reflex bradycardia, as reflected by the essentially unchanged RPP from control. The expected effect of increased cardiac afterload with elevated Pa became apparent when HR was restored to control levels during phenylephrine infusion. In fact, BMR was markedly reduced compared to PE alone, enabling an increase in coronary blood flow to meet the higher metabolic demand. These physiological responses to perturbations of coronary and systemic hemodynamics underlie potential changes in functional indices of stenosis severity assessed at baseline.

### Effects of changes in Pa and HR on basal stenosis indices and diagnostic classification

We are not aware of other publications that reported changes in basal stenosis indices caused by altered metabolic demand in humans. In our study, the pressure-only index Pd/Pa was more prone to be affected by alterations in resting coronary blood flow than BSR, which combines pressure and velocity measurements. During tachycardia, close to one-third of the lesions switched diagnostic classification compared to control according to the Pd/Pa binary threshold. BSR tended to increase with pacing, similar to prior findings in dogs [13], but statistical significance was not achieved in our study.

We could not derive iFR, but would expect it to be similarly affected as whole-cycle or diastolic Pd/Pa by changes in metabolic state at rest.

In general, baseline indices to identify the ischemic potential of coronary lesions suffer from a higher misclassification rate compared with established hyperemic indices [11, 15, 17]. This can partly be attributed to the low magnitude of intracoronary signals obtained at resting flow, which amplifies the relative effect of measurement errors due to pressure sensor drift (Pd), hydrostatic pressure offset (Pa), or a lower signal-to-noise ratio (velocity) [4, 20, 31, 38] and has prompted some investigators to consider a decision-making strategy based on resting pressures a sub-optimal approach [11, 16]. Since in general, the factors contributing to variability at baseline are relatively less potent at higher flow, contrast- or adenosine induced elevated flow is advisable and may also mitigate effects of altered metabolic state at the time of measurement.

### Study limitations

We recognize that the small cohort of patients limits extension of our findings to a more general population. However, our results were consistent with physiological expectations and warrant further research on this topic in a larger group of patients.

The range and distribution of stenosis severities have been identified as a critical factor in terms of diagnostic accuracy for baseline indices [15]. Our re-classification rate following hemodynamic alterations should, therefore, be interpreted in this context and not be considered exemplary for a wider population. In addition, the heterogeneous distribution of stenosis severities in a small number of patients may also have adversely affected the significance of differences between the explored variables.

We gave phenylephrine intravenously to increase aortic pressure by increasing peripheral vasoconstriction. Phenylephrine may have stimulated α_1_-adrenergic receptors in epicardial coronary vessels [12]. In this respect, the absence of observable vasodilation during increased afterload (PE) may also have been related to α-adrenergic coronary vasoconstriction. However, we believe this effect to be minimal, since potential α-mediated vasoconstriction is likely overruled by metabolic demand [3, 18] and coronary flow velocity has been shown not to change during α-adrenergic blockade with phentolamine or urapidil compared to phenylephrine alone [21, 34].

Confounding factors such as co-morbidities, medication, or microvascular disease can affect autoregulation [5, 12, 22]. Our study group is too small to allow a methodological comparison between subgroups. Moreover, in our study, each stenosis served as its own control. Therefore, we do not expect these factors to alter the general message of our study.

We did not measure absolute flow, but flow velocity, since this is the clinically available signal for intracoronary measurements. Consequently, we derived velocity-based resistance indices. In contrast to volume flow, flow velocity is much less dependent on perfusion territory, since it is preserved from proximal to distal segments in normal coronary arteries [28]. Sensor location was unchanged throughout the protocol steps for each patient. Nitroglycerine was given at the start of the protocol to minimize variations in vessel diameter due to changes in vascular tone at the measurement site [33]. Therefore, since the effect of interventions was expressed in terms of intra-subject comparisons to the respective control value, we do not expect the individual size of perfusion areas to differentially affect control and intervention measures or, consequently, our overall findings.

Although the magnitude of hemodynamic provocations produced in this study is not likely to be encountered during regular catheterization procedures, they demonstrate the potential for intrinsic biological variability in resting coronary flow to affect baseline stenosis indices.

## Conclusion

Systemic hemodynamic perturbations caused resting flow velocity and BMR to adapt to altered myocardial metabolic demand. Consequently, basal indices of stenosis severity were affected, resulting in diagnostic re-classification according to Pd/Pa and BSR thresholds. The results of future investigations should, therefore, be examined with attention to determinants of myocardial oxygen demand when assessing baseline indices of stenosis severity.
